# Supplementary material for: Effect of trade on global aquatic food consumption patterns
Source: Nat Commun. 2024 Feb 15;15:1412. doi: 10.1038/s41467-024-45556-w (PMC10869811; doi:10.1038/s41467-024-45556-w)
Supplement: Supplementary file 1 — Supplementary Information [file 41467_2024_45556_MOESM1_ESM.pdf]

Supplementary Information for

Effect of trade on global aquatic food consumption patterns

Kangshun Zhao, Steven D. Gaines, Jorge García Molinos, Min Zhang, Jun Xu

Global aquatic food production patterns

Production trends. Over the past decades, global aquaculture and capture fisheries production increased, especially for aquaculture (Supplementary Fig. 1). All continents' aquaculture production has increased. Asia has seen a considerable increase in both aquaculture and capture fisheries, with aquaculture in particular growing rapidly. China is the world's largest producer and has contributed the most to the growth of global aquaculture production over the past decades (Supplementary Fig 3a-d). Although aquaculture development in Europe and North America was rapid during the 1980s-1990s but has since stagnated, probably owing to regulatory restrictions on sites and other competitive factors, although markets for aquatic foods have continued to grow[1]. Except for Asia, the rest of the world occupies a small share of aquaculture production. In addition to Asia, Europe and South America are also one of the world's two main fishing areas, and catch has been declining since the 1990s. Since we only considered the species for direct human consumption, we excluded the world's most important forage fish (i.e., anchoveta) from the captured dataset. Therefore, the capture fisheries production in South America was largely underestimated. From 1976 to 2019, the capture fisheries production in Africa increased slightly, stable in North America and Oceania. The mean per capita production of aquatic foods in Africa is lowest and highest in Oceania from 1976 to 2019 (Fig. 3 and Supplementary Fig. 5a).

Trophic level trends. The global trophic level of capture fisheries was generally stable except for South America and Oceania, which showed an upward trend (Supplementary Fig. 2). Compared to aquaculture, the global and continental mean trophic levels of capture fisheries are almost unchanged. The mean trophic level of aquaculture has increased in Europe, South America, and Oceania, with no apparent trend in Asia and Africa, and decreased in North America after 2000. In Europe, North America, and Oceania, where there are more developed countries, the mean trophic level of aquaculture is higher than that of capture fisheries. While the mean trophic level of aquaculture in Asia, Africa, and South America, where most of the

developing countries are located, is lower than that of capture fisheries (Supplementary Fig. 2). There was little difference in the capture trophic level, while it was great in aquaculture trophic level between different countries and regions (Supplementary Fig. 3e, f). The rapid rise in aquaculture trophic levels in Europe, South America, and Oceania indicates that there has been an increasing emphasis on the cultivation of high-trophic level species in recent decades, especially in Europe. The decline in aquaculture trophic levels in North America over the last 20 years indicates a rapid increase in the production of low-trophic level species, especially in Mexico. In recent decades, Asia has been the largest producer of low-trophic level species, especially China (Supplementary Figs. 2, 3, and 5b). Currently, Asia dominates world aquaculture, producing 91.6% of the total[2].

Supplementary Tables and Figures

Supplementary Tables

Table 1. Top 20 countries in mean import and export volume from 1976 to 2019

Country	Imports (tonnes)	Percentage of total import volume	Country	Exports (tonnes)	Percentage of total export volume
USA	108798275	11%	China	128152290	13%
Japan	104569389	10%	Norway	73857890	7%
China	74402497	7%	Thailand	73518692	7%
Spain	49474738	5%	USA	50298087	5%
France	45171623	4%	Russia	38966398	4%
Germany	45102509	4%	Denmark	33413276	3%
Italy	38755985	4%	Netherlands	33405349	3%
Thailand	38314615	4%	Spain	31833176	3%
UK	37331901	4%	Vietnam	30897318	3%
Nigeria	32438213	3%	Canada	29698585	3%
South Korea	30474150	3%	Indonesia	26751268	3%
Netherlands	28154403	3%	Iceland	26001328	3%
Denmark	27334135	3%	UK	25585938	3%
Russia	23981597	2%	India	24183390	2%
Portugal	17688443	2%	Germany	22604032	2%
Sweden	17250123	2%	Japan	21533233	2%
Canada	16144385	2%	Chile	20985870	2%
Malaysia	15725537	2%	South Korea	18734204	2%
Poland	14586701	1%	Morocco	17934184	2%
Brazil	11693045	1%	Argentina	16814699	2%
Total		75%	Total		75%

Table 2. The proportion of countries affected by trade in each region and globally

**Region**	**Positive (%)**	**Negative (%)**	**Unchanged (%)**
**HATL**			
Africa	71.7%	28.3%	0.0%
Asia	68.2%	31.8%	0.0%
Europe	73.7%	26.3%	0.0%
North America	52.4%	47.6%	0.0%
Oceania	8.3%	91.7%	0.0%
South America	69.2%	30.8%	0.0%
Globally	64.4%	35.6%	0.0%
**Aquatic food consumption per capita**			
Africa	78.3%	21.7%	0.0%
Asia	65.9%	34.1%	0.0%
Europe	73.7%	26.3%	0.0%
North America	57.1%	42.9%	0.0%
Oceania	50.0%	41.7%	8.3%
South America	30.8%	69.2%	0.0%
Globally	66.1%	33.3%	0.6%

Note: Each country's positive or negative impacts were identified by mean change in annual HATL or per capita consumption of aquatic foods after trade from 1976 to 2019. Population data was mainly from the World Bank's database (https://datacatalog.worldbank.org/search/dataset/0037712/World-Development-Indicators), several countries' population data (French Guiana, Guadeloupe, Kuwait, Martinique, Mayotte, Palestine, and Reunion) was from the United Nations World Population Prospects 2022 revision[3]. HATL= the human aquatic food trophic level.

Table 3. Detailed matching information between several export commodity groups and production items

Export commodity groups	Matching production items
Flatfish	Halibut
	Dab
	Flounder
	Plaice
	Turbot
	Megrim
	Sole
Salmonids	Trout
	Char
	Salmon
Clupeidae	Herring
	Shad
	Sardine
	Menhaden
	Pilchard
	Sprat
Euthynnus other than skipjack	Kawakawa
	Little tunny

Table 4. Summary of the conversion factors for different preserved aquatic products to whole-animal live weight

Taxon	Portion/form	Preprocessing factor	Preservation method	Preservation factor
Crustacean	Shrimp/Prawn/Lobster/Crayfish (Meat or tails)	1.43	Fresh/chilled/frozen	1
	Shrimp/Prawn/Lobster/Crayfish (In shell or not)	1.21[1]	Prepared/preserved	2.27[2]
	Crab (In shell or not)	1.82[1]	Dried/salted	2.54
	Crab (Meat)	2.63	Smoked	2
Fish	Gutted/Heads-off/Loins/Fillets/Meat/Minced/ Flaps/Flakes/Sides/Steaks/Sticks	1.25		
	Heads/tail/maws	5		
Cephalopod	Rings	1.25		
**Taxon**	**Product**			**Overall factor**
Fish	Dried/Salted fish			2.82
	Smoked fish			2.5
	Prepared/preserved fish			2.66[3]
	Fish paste			0.88
	Fish sauce			0.5
Cephalopod	Dried/Salted cephalopod			2.82
	Smoked cephalopod			2.5
	Prepared/preserved cephalopod			2.66[3]

Note: [1]Mean of preprocessing factor for shelled and unshelled; [2]Mean of preservation factor for dried/salted and smoked preservation method; [3]Mean of the overall factor for dried/salted and smoked fish or cephalopod. The preprocessing factor of different crustaceans was sourced from Hortle [4]. The overall conversion factor for cephalopods was considered to be the same as that for fish. The preservation factor of crustaceans and cephalopods was considered to be the same as that of fish, which assumed that protein content is equal.

Supplementary Figures

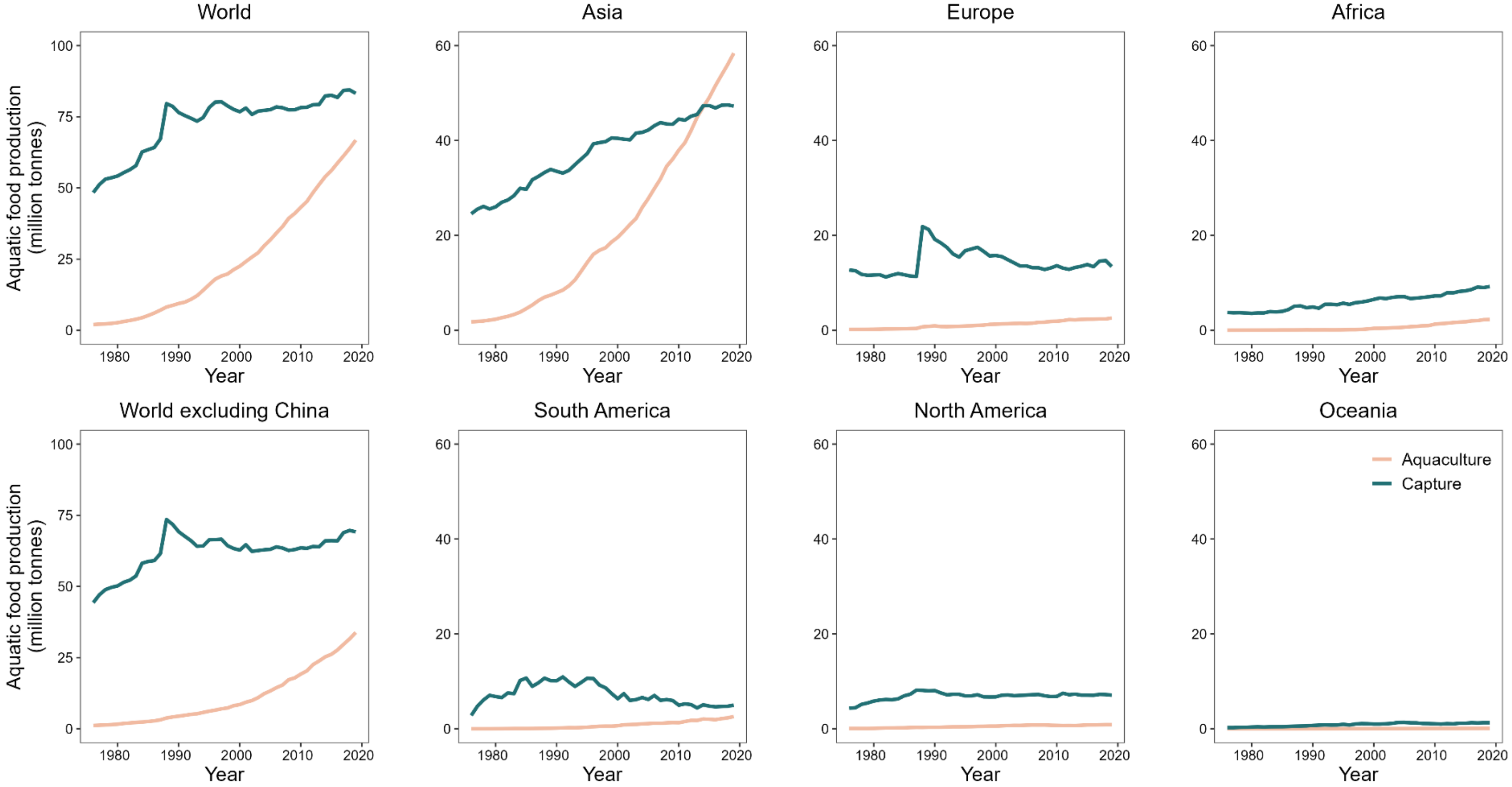

Figure 1. Global aquatic food production of aquaculture and capture fisheries from 1976 to 2019.

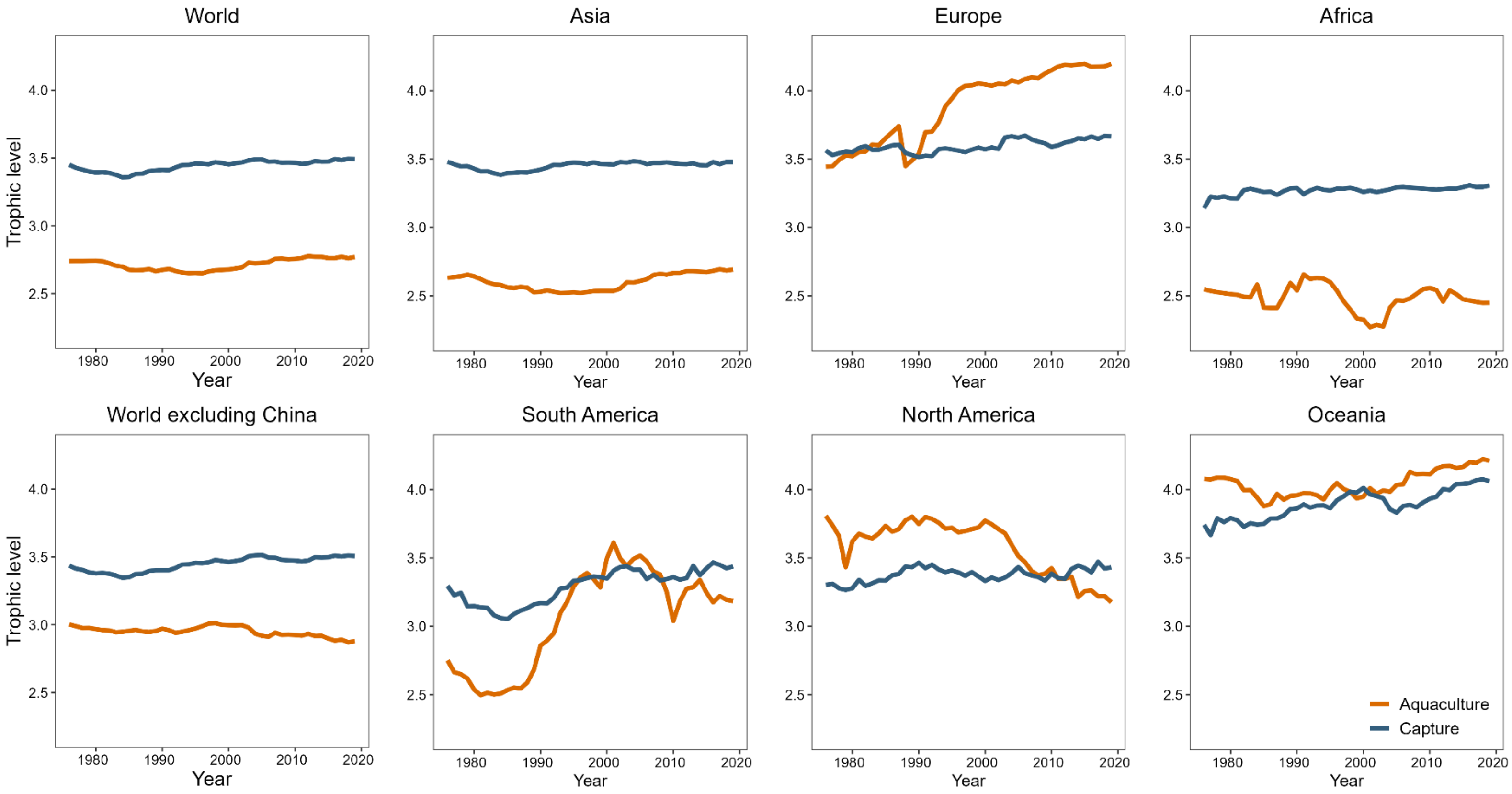

Figure 2. Global mean trophic level of aquaculture and capture fisheries from 1976 to 2019.

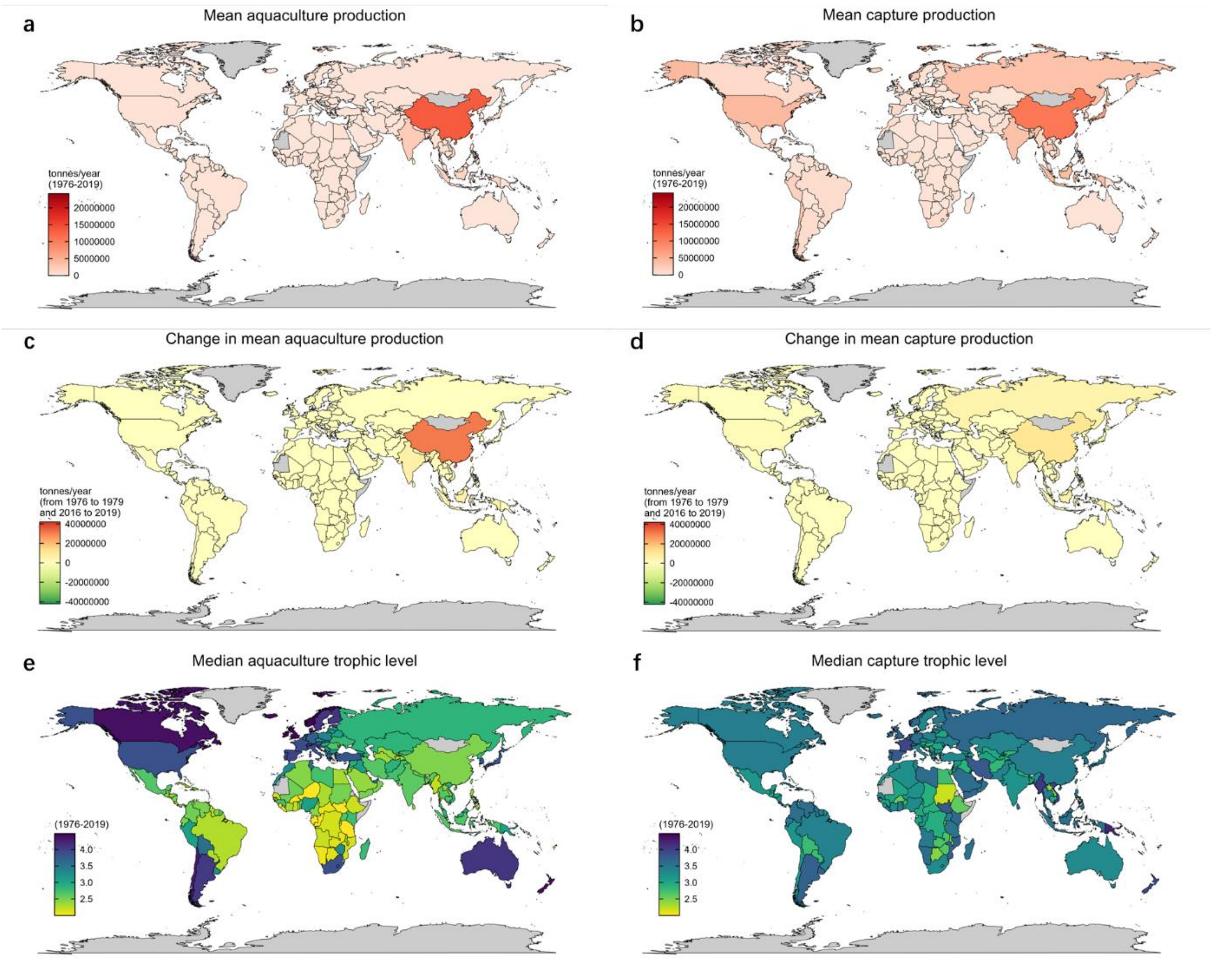

Figure 3. Global trophic level and production of aquaculture and capture fisheries. a-b, The country-level mean production of aquaculture (**a**) and capture fisheries (**b**). **c-d,** The change in country-level mean production of aquaculture (**c**) and capture fisheries (**d**) from 1976 to 1976 and 2015 to 2019. **e-f,** The country-level median trophic level of aquaculture (**e**) and capture fisheries (**f**).

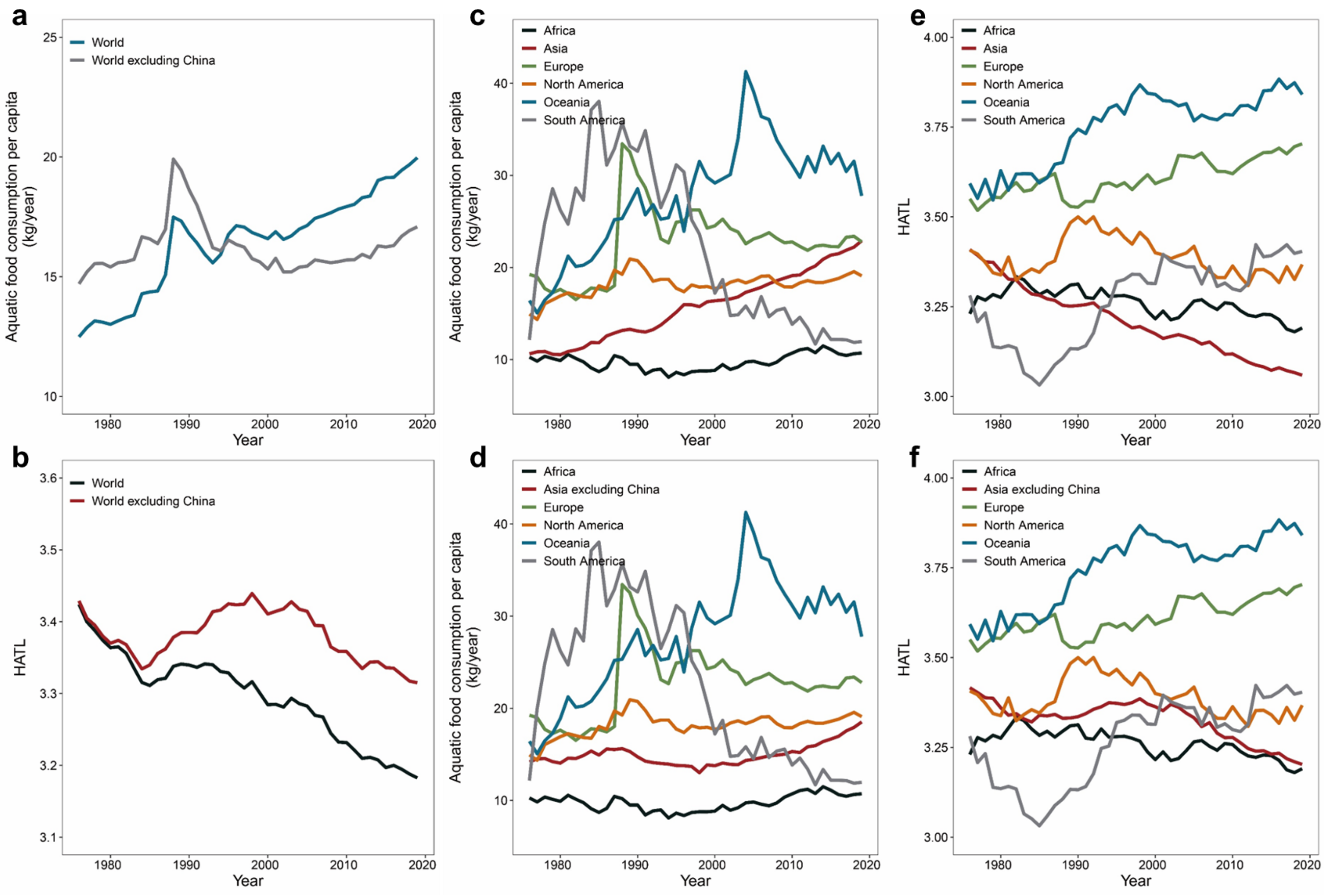

Figure 4. Comparisons of global trends of HATL and per capita consumption of aquatic foods when excluding China from 1976 to 2019. a-b, The global trends of per capita consumption of aquatic foods **(a)** and HATL **(b)**. **c-d**, The continental trends of per capita consumption of aquatic foods including **(c)** and excluding China **(d)**. **e-f**, The continental trends of HATL including **(e)** and excluding China **(f)**.

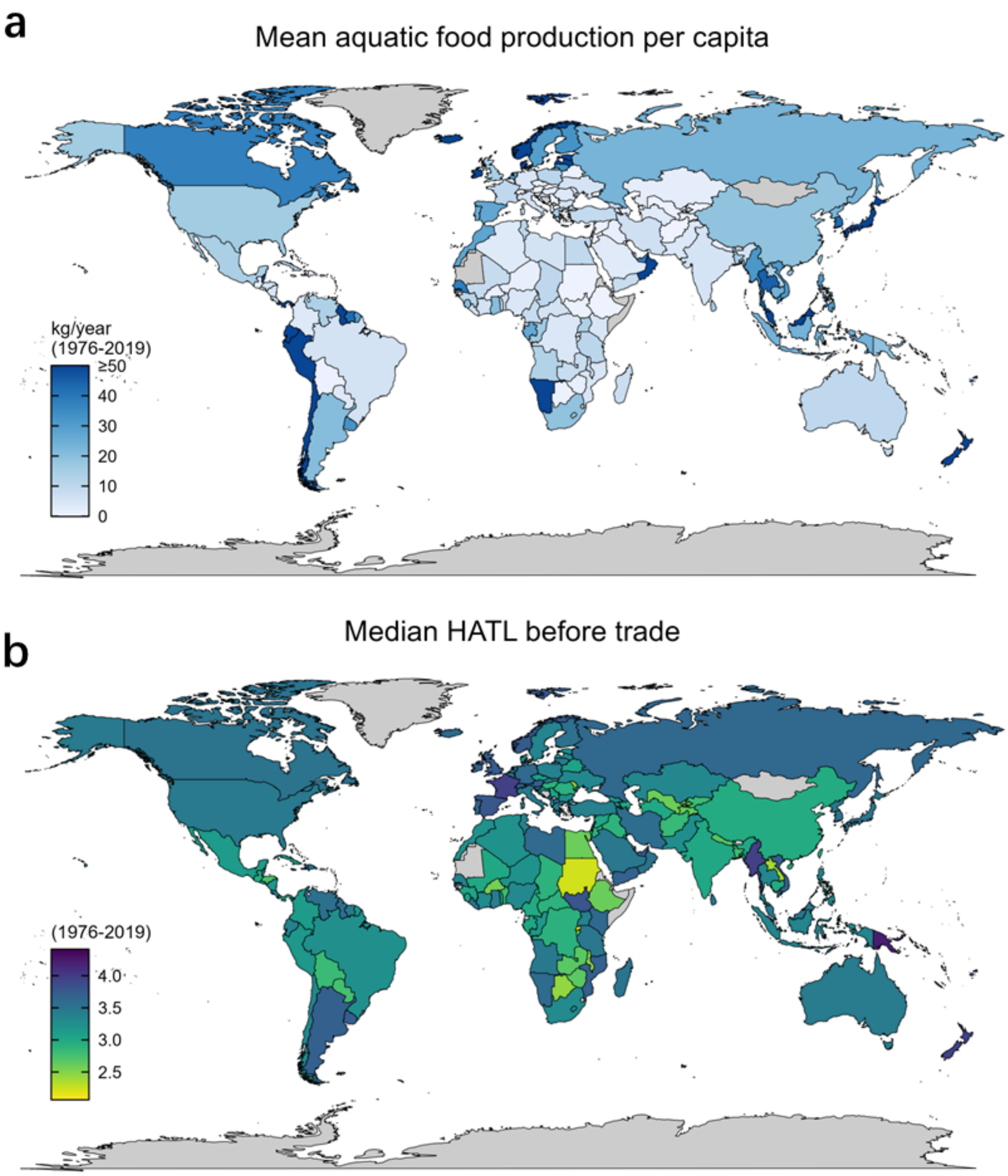

Figure 5. Global aquatic food production and HATL before trade from 1976 to 2019. a, The mean per capita production of aquatic foods. **b**, The median country-level HATL before trade.

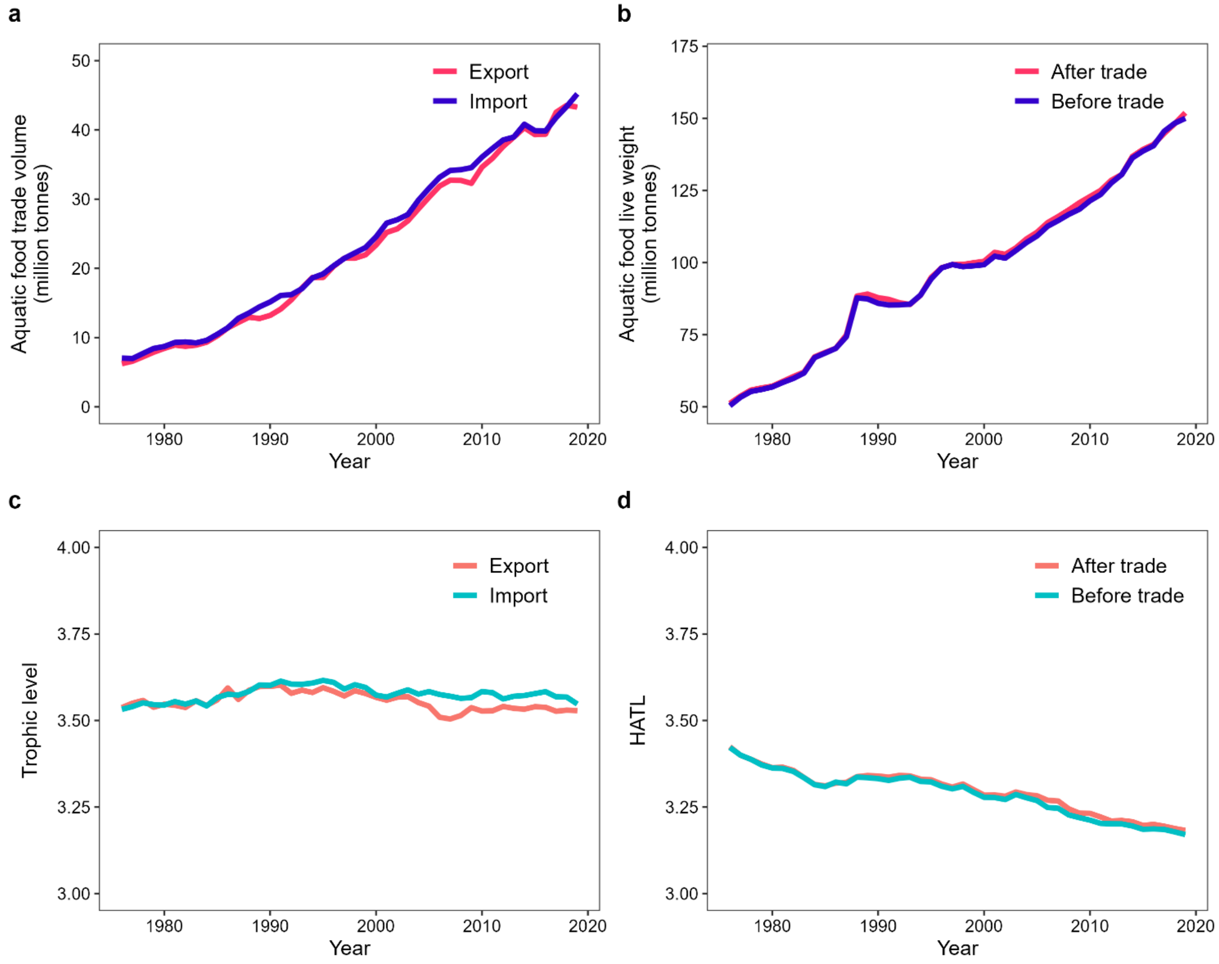

Figure 6. Mass balance rationality analysis. a, Global aquatic food trade volume of imports and exports (theoretical exports). **b**, Global aquatic food live weight trends before and after trade. **c**, Global mean trophic level trends of imports and exports. **d**, Global human aquatic food trophic level trends before and after trade.

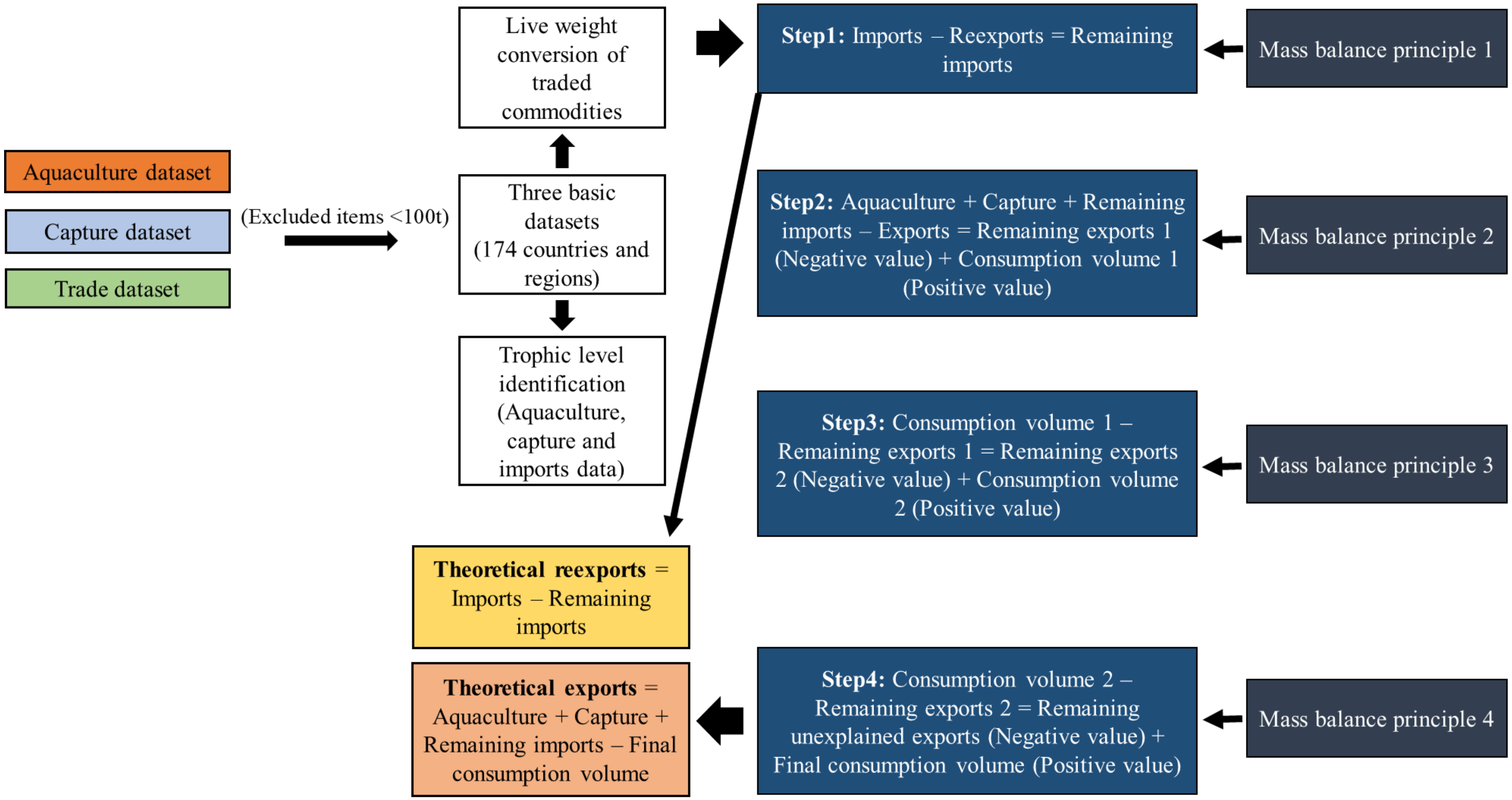

Figure 7. Workflow of species-level mass balance from FAO statistics

References

1 Bostock, J. *et al.* Aquaculture: global status and trends. *Philosophical Transactions of the Royal Society B: Biological Sciences* **365**, 2897-2912 (2010).

2 FAO. *The State of World Fisheries and Aquaculture*. Vol. 4 (FAO, 2022).

3 Nations, U. (Department of Economic and Social Affairs, Population Division, 2022).

4 Hortle, K. G. Consumption and the yield of fish and other aquatic animals from the Lower Mekong Basin. 1-88 (Mekong River Commission, Vientiane, Lao PDR, 2007).
