# Supplementary material for: Effect of trade on global aquatic food consumption patterns
Source: Nat Commun. 2024 Feb 15;15:1412. doi: 10.1038/s41467-024-45556-w (PMC10869811; doi:10.1038/s41467-024-45556-w)
Supplement: Supplementary file 3 — Description of Additional Supplementary Files [file 41467_2024_45556_MOESM3_ESM.pdf]

Description of Additional Supplementary Files

File Name: Supplementary Data 1
Description: Live weight and conversion factors of traded commodities

File Name: Supplementary Data 2
Description: Trophic level identification and production data

File Name: Supplementary Data 3
Description: Detailed information for trophic level identification
