# Supplementary material for: Effect of trade on global aquatic food consumption patterns
Source: Nat Commun. 2024 Feb 15;15:1412. doi: 10.1038/s41467-024-45556-w (PMC10869811; doi:10.1038/s41467-024-45556-w)
Supplement: Supplementary file 7 — Reporting Summary [file 41467_2024_45556_MOESM7_ESM.pdf]

nature portfolio

Corresponding author(s): Jun Xu and Min Zhang

Last updated by author(s): Nov 23, 2023

Reporting Summary

Nature Portfolio wishes to improve the reproducibility of the work that we publish. This form provides structure for consistency and transparency in reporting. For further information on Nature Portfolio policies, see our Editorial Policies and the Editorial Policy Checklist.

Statistics

For all statistical analyses, confirm that the following items are present in the figure legend, table legend, main text, or Methods section.

n/a	Confirmed	
☐	☒	The exact sample size (*n*) for each experimental group/condition, given as a discrete number and unit of measurement
☒	☐	A statement on whether measurements were taken from distinct samples or whether the same sample was measured repeatedly
☒	☐	The statistical test(s) used AND whether they are one- or two-sided<br>*Only common tests should be described solely by name; describe more complex techniques in the Methods section.*
☒	☐	A description of all covariates tested
☒	☐	A description of any assumptions or corrections, such as tests of normality and adjustment for multiple comparisons
☒	☐	A full description of the statistical parameters including central tendency (e.g. means) or other basic estimates (e.g. regression coefficient) AND variation (e.g. standard deviation) or associated estimates of uncertainty (e.g. confidence intervals)
☒	☐	For null hypothesis testing, the test statistic (e.g. *F*, *t*, *r*) with confidence intervals, effect sizes, degrees of freedom and *P* value noted<br>*Give P values as exact values whenever suitable.*
☒	☐	For Bayesian analysis, information on the choice of priors and Markov chain Monte Carlo settings
☒	☐	For hierarchical and complex designs, identification of the appropriate level for tests and full reporting of outcomes
☒	☐	Estimates of effect sizes (e.g. Cohen's *d*, Pearson's *r*), indicating how they were calculated

Our web collection on statistics for biologists contains articles on many of the points above.

Software and code

Policy information about availability of computer code

Data collection	Three original global fisheries statistic datasets were taken from the FishStatJ software (Version 2021). The trophic level of specific species was extracted directly from Fishbase (www.fishbase.org). The conversion factors were taken from literatures (i.e., Hortle 2007 and Fluet-Chouinard, Funge-Smith et al. 2018).
Data analysis	We subtracted export weights from production in four custom sequential steps. All statistical analyses were performed using R version 4.1.1 for Windows. The R codes used to conduct the study are available in the GitHub (https://github.com/zhaokangshun/Effect-of-trade-on-global-aquatic-food-consumption-patterns.git) and archived through Zenodo (https://doi.org/10.5281/zenodo.10129727).

For manuscripts utilizing custom algorithms or software that are central to the research but not yet described in published literature, software must be made available to editors and reviewers. We strongly encourage code deposition in a community repository (e.g. GitHub). See the Nature Portfolio guidelines for submitting code & software for further information.

Data

Policy information about availability of data

All manuscripts must include a data availability statement. This statement should provide the following information, where applicable:

- Accession codes, unique identifiers, or web links for publicly available datasets
- A description of any restrictions on data availability
- For clinical datasets or third party data, please ensure that the statement adheres to our policy

The raw data used in this study are publicly available (see Methods). The processed data generated in this study are either included in the Supplementary Information or available in the figshare repository (https://doi.org/10.6084/m9.figshare.21692186.v3).

Research involving human participants, their data, or biological material

Policy information about studies with human participants or human data. See also policy information about sex, gender (identity/presentation), and sexual orientation and race, ethnicity and racism.

Reporting on sex and gender	Not Applicable.
Reporting on race, ethnicity, or other socially relevant groupings	Not Applicable.
Population characteristics	Not Applicable.
Recruitment	Not Applicable.
Ethics oversight	Not Applicable.

Note that full information on the approval of the study protocol must also be provided in the manuscript.

Field-specific reporting

Please select the one below that is the best fit for your research. If you are not sure, read the appropriate sections before making your selection.

☐ Life sciences ☒ Behavioural & social sciences ☐ Ecological, evolutionary & environmental sciences

For a reference copy of the document with all sections, see nature.com/documents/nr-reporting-summary-flat.pdf

Behavioural & social sciences study design

All studies must disclose on these points even when the disclosure is negative.

Study description	The study aims to clarify the global characteristics of aquatic food consumption and trade, as well as the impact of trade in different countries and regions, by developing a species-level mass balance dataset and a trophic level identification dataset for 174 countries, using quantitative data.
Research sample	FishStatJ is a Java-based desktop application which provides users with access to a variety of global fishery statistical datasets from FAO. It consists of a main application and several workspaces that include the datasets.
Sampling strategy	Not Applicable.
Data collection	All data acquired from public software (i.e., FishStatJ software), website (www.fishbase.org) and related literatures (i.e., Hortle 2007 and Fluet-Chouinard, Funge-Smith et al. 2018).
Timing	From October 2021 to June 2022.
Data exclusions	We removed all items of negligible importance (i.e., total volume <100t) from 1976 to 2019 in all original datasets from the FishStatJ software to facilitate the definition of the live weight conversion factor for trade commodities and the trophic level of species or species groups in aquaculture and capture datasets according to the fishing area in each country. Removed items accounted for a total of 0.001% of aquaculture production, 0.004% of capture production, and 0.044% of trade volume. To determine whether the removal of production and trade items with a total volume < 100t from 1976 to 2019 would result in highly unequal impacts on small and large countries, we examined the proportion of removed items in production and trade for all 174 countries. We discovered that in 97% of countries, the ratio of excluded items to total production is below 1%. Additionally, in 95% of countries, the proportion of excluded items to total trade volume is less than 3%. Therefore, the removal of items with a total volume <100t would not have significant unequal impact on different countries.

Non-participation	Not Applicable.
Randomization	Not Applicable.

Reporting for specific materials, systems and methods

We require information from authors about some types of materials, experimental systems and methods used in many studies. Here, indicate whether each material, system or method listed is relevant to your study. If you are not sure if a list item applies to your research, read the appropriate section before selecting a response.

Materials & experimental systems

n/a	Involved in the study
☒	☐ Antibodies
☒	☐ Eukaryotic cell lines
☒	☐ Palaeontology and archaeology
☒	☐ Animals and other organisms
☒	☐ Clinical data
☒	☐ Dual use research of concern
☒	☐ Plants

Methods

n/a	Involved in the study
☒	☐ ChIP-seq
☒	☐ Flow cytometry
☒	☐ MRI-based neuroimaging

Plants

Seed stocks	Not Applicable.
Novel plant genotypes	Not Applicable.
Authentication	Not Applicable.
